# Was Antiphospholipid Syndrome a Risk Factor of Stroke? A Systemic Review and Meta-Analysis of Cohort Studies

**DOI:** 10.1155/2021/4431907

**Published:** 2021-12-16

**Authors:** Kai Zhao, Ping Zhou, Ling Xu, Ruili Li, Jincai Yang, Qiang Zhang, Mingfei Yang, Xiaoxing Wei

**Affiliations:** ^1^Graduate School, Qinghai University, Xining, Qinghai 810016, China; ^2^Department of Neurosurgery, Qinghai Provincial People's Hospital, Xining, Qinghai 810007, China; ^3^Department of Community Health Education, Institute for Health Education of Qinghai Province, Xining, Qinghai 810000, China; ^4^Department of Neurological Intensive Care, Shengli Oilfield Central Hospital, Dongying, Shandong 257000, China; ^5^Medical College of Qinghai University, Xining, Qinghai 810016, China

## Abstract

Antiphospholipid syndrome (APS) is characterized by thrombosis. This systemic review and meta-analysis was to verify the hypothesis that APS might increase the risk of stroke. Studies were identified after literature searching of PubMed, Embase, and Cochrane. Newcastle-Ottawa Quality Assessment Scale Cohort Studies (NOQAS-C) was used to assess the quality of studies. The pooled effect with 95% confidence interval (95% CI) was calculated by random-effect model. *I*-square (*I*^2^) was used to test heterogeneity. Funnel plot was used to evaluate publication bias. A total of 17 cohort studies with overall high quality were included. There was no publication bias. Pooled hazard ratio of stroke occurrence in APS patients was 1.76 (1.39-2.21) with low heterogenicity and stable result from sensitivity analysis. In the analysis of subgroups, pooled risk ratios of stroke occurrence in patients with only positive antibodies of APS diagnosis were 1.75 (0.99-3.09), which for the APS patients with other autoimmune diseases were 14.70 (7.56-28.56). APS might be a risk factor of stroke, especially in patients with other autoimmune diseases.

## 1. Introduction

Antiphospholipid syndrome (APS) is a disease characterized by recurrent arterial or venous thrombosis, pathological pregnancy, and consistently positive antiphospholipid antibodies (such as lupus anticoagulant laC, anticardiopholipid antibody aCA, and anti-*β*_2_GP1 antibody a*β*A) [1]. APS could not only be secondary to the systemic lupus erythematosus (SLE) or other autoimmune diseases but also occur alone (known as primary APS (PAPS)) [2]. The incidence of APS is significantly higher in females than in males, and in particular, some females first find APS when they discover the causes of spontaneous miscarriage [3]. Due to consistently positive antiphospholipid antibodies, the abnormal coagulation function and vascular endothelial injury could bring about thrombosis events in many organs, leading to stroke, myocardial infarction, limb ischemia, and so on [4]. In addition to ischemic stroke (ISS), the platelet reduction can also appear in APS patients, which could be associated to hemorrhagic diseases, including the pulmonary alveolar hemorrhage and the intracranial hemorrhage (ICH) [5, 6]. However, in evidence-based medicine, there is still no convincing evidence supporting that APS increases the risk of stroke. Moreover, it is of great significance to find out whether patients with only positive antibody of APS diagnosis would face the onset risk. Therefore, this systemic review and meta-analysis based on cohort studies was performed to verify the hypothesis that APS might be the risk factor of stroke.

## 2. Methods

This systemic review and meta-analysis was performed referring to the protocol published on the database of International Platform of Registered Systematic Review and Meta-analysis Protocols (INPLASY, https://inplasy.com/, registration number: INPLASY202180074, DOI number: 10.37766/inplasy2021.8.0074).

### 2.1. Data Sources

Literature searching was performed in three public electronic databases of PubMed, Embase, and Cochrane. The strategy of literature searching was as follows: (((((((((((((((((((“Stroke”/exp) OR (“Stroke∗”:ab,ti)) OR (“Cerebrovascular Disease∗”:ab,ti)) OR (“CVA∗ (Cerebrovascular Accident)”:ab,ti)) OR (“Cerebrovascular Apoplexy”:ab,ti)) OR (“Apoplexy, Cerebrovascular”:ab,ti)) OR (“Vascular Accident, Brain”:ab,ti)) OR (“Brain Vascular Accident∗”:ab,ti)) OR (“Vascular Accidents, Brain”:ab,ti)) OR (“Cerebrovascular Stroke∗”:ab,ti)) OR (“Stroke∗, Cerebrovascular”:ab,ti)) OR (“Apoplexy”:ab,ti)) OR (“Cerebral Stroke∗”:ab,ti)) OR (“Stroke∗, Cerebral”:ab,ti)) OR (“Stroke, Acute”:ab,ti)) OR (“Acute Stroke∗”:ab,ti)) OR (“Strokes, Acute”:ab,ti)) OR (“Cerebrovascular Accident∗, Acute”:ab,ti)) OR (“Acute Cerebrovascular Accident∗”:ab,ti)) AND (((((((((((((((“Antiphospholipid Syndrome”/exp) OR (“Syndrome, Antiphospholipid”:ab,ti)) OR (“Hughes Syndrome”:ab,ti)) OR (“Syndrome, Hughes”:ab,ti)) OR (“Antiphospholipid Antibody Syndrome”:ab,ti)) OR (“Antibody Syndrome, Antiphospholipid”:ab,ti)) OR (“Antiphospholipid Antibody Syndromes”:ab,ti)) OR (“Syndrome, Antiphospholipid Antibody”:ab,ti)) OR (“Anti-Phospholipid Antibody Syndrome”:ab,ti)) OR (“Anti Phospholipid Antibody Syndrome”:ab,ti)) OR (“Antibody Syndrome, Anti-Phospholipid”:ab,ti)) OR (“Syndrome, Anti-Phospholipid Antibody”:ab,ti)) OR (“Anti-Phospholipid Syndrome”:ab,ti)) OR (“Anti Phospholipid Syndrome”:ab,ti)) OR (“Syndrome, Anti-Phospholipid”:ab,ti)).

### 2.2. Study Selection

Inclusion criteria are as follows: (1) language, regions, and publication years of articles were not restricted; (2) cohort studies; (3) participants in the exposure group suffered from APS; (4) participants in the nonexposure group only differed in no APS suffering; (5) endpoint of observation was stroke; and (6) analysis of cohort studies' outcomes was completely performed. Exclusion criteria are as follows: (1) duplication; (2) reviews, comments, letters, case reports, protocols of clinic trials, or conference papers; (3) animal experiments; and (4) contents of articles were irrelevant to this meta-analysis.

### 2.3. Quality Assessment of Studies

The quality assessment of included articles was performed via the Newcastle-Ottawa Quality Assessment Scale Cohort Studies (NOQAS-C) before data extraction. We considered that the studies with larger size of included patients should be assessed to have higher quality, in accordance with the results from the NOQAS-C assessment.

### 2.4. Data Extraction

All data used to assess the outcomes of these cohort studies were extracted, including the hazard ratio (HR), risk ratio (RR), and odds ratio (OR). In addition, some confounders, which might result in errors, were adjusted, including the ages, gender, accompanying conditions of participation before study, definition of endpoint, and period of observation.

### 2.5. Statistical Methods

Relative numbers and their 95% confidence intervals (95% CI) were used to describe the count data. Meta-analysis was performed using corresponding modules with the Software for Statistics and Data Science (Stata, version 15.1; College Station, Texas 77845 USA). The pooled effect with its 95% CI was calculated by the random-effect model. The *I*-square (*I*^2^) was used to test the heterogeneity. Sensitivity analysis was performed to evaluate the stability of the overall results by recalculating the pooled effects of the remaining studies after omitting the highest-quality study, or the random-effect model was switched to the fixed-effect model. Funnel plot symmetry or Egger's regression was used to evaluate the publication bias. To reduce heterogeneity, the pooled effects of the remaining studies would be recalculated after omitting the lowest-quality study, or the subgroup analysis was performed directly. All *p* values were two-sided with a significant level at 0.05.

## 3. Results

### 3.1. Selection

**Figure 1:**
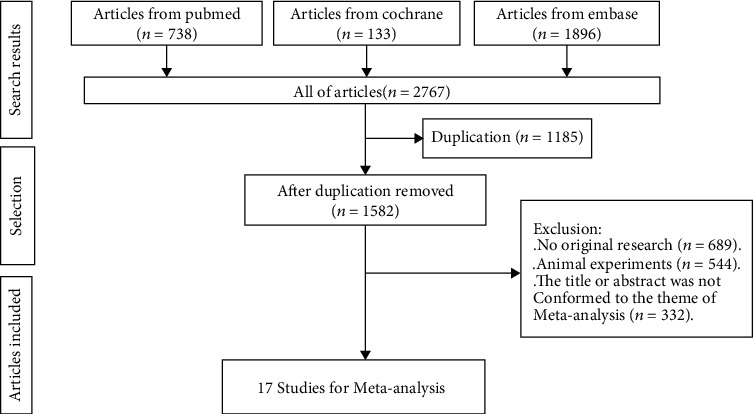
Process of literature search.

In total, 2767 articles were retrieved from the databases according to the study strategy. After screening based on the inclusion and exclusion criteria, 17 cohort studies [7–23] were enrolled ultimately (Figure 1). There were a total of 7144 people participating in all these studies, including 1289 males (Table 1). The age range was 6 to 72 years old. Countries or regions involved Asia, America, and Europe. Publication years were from 1993 to 2021, including all the cohort studies in the 21^st^ century. Periods of observation were from 2 to 38 years. There were also other concomitant characters of participation before studies, which might influence results of our meta-analysis. These confounding factors could be divided into 3 major groups: (1) none; (2) other concomitant autoimmune diseases, including SLE, rheumatoid arthritis (RA), and related organ injuries (such as heart injuries); and (3) APS diagnosis absolutely depended on different types of positive antibodies, which meant that the APS patients were divided into the following: (1) the single positive antibody groups, such as laC(+), aCA(+), and a*β*A(+); (2) double positive antibody group, such as laC/aCA(+), aCA/a*β*A(+), and laC/a*β*A(+); and (3) triple positive antibody group. In addition, one study defined the endpoint that patients suffered from transient ischemic attack (TIA) or ISS, which were subtypes of stroke. Therefore, considering these factors, different data might be extracted from the same article.

### 3.2. Comparability

According to NOQAS-C, three studies (2018Serena Fasano, 2013Chi Chiu Mok, and 2020Aline G. Islabão) were scored as 9, which was the highest score (Table 2). In the Selection section, three articles were assessed to be with high risk of the nonexposed segment, and six articles were assessed to be with high risk of the not present segment. Five articles were assessed to be with risk in the Comparability section. In the Outcome section, 4 articles were assessed to be with high risk of the long enough segment, and 9 articles were assessed to be with high risk of the adequacy segment. Finally, 3 articles were scored as 6 (2016Jean-Christophe Gris, 2018Radin Massimo, and 2019Kanon Jatuworapruk), which was the lowest score.

### 3.3. Outcome

**Figure 2:**
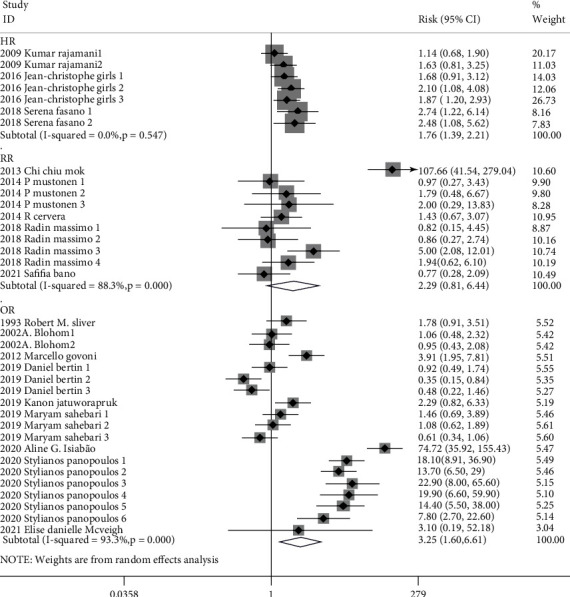
Totally pooled HR/RR/OR.

**Figure 3:**
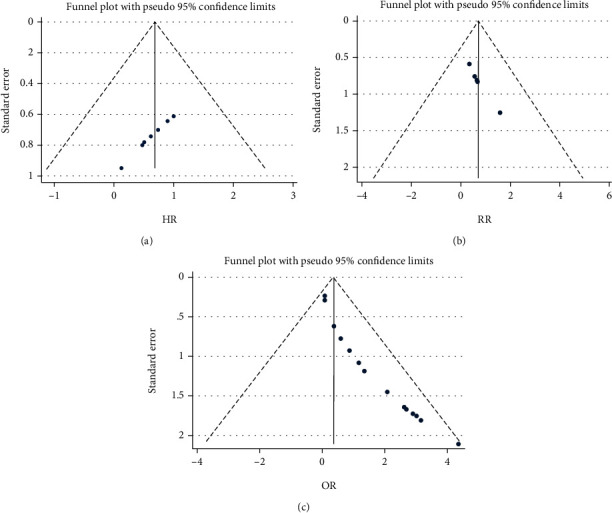
Funnel plots of totally pooled HR/RR/OR.

**Figure 4:**
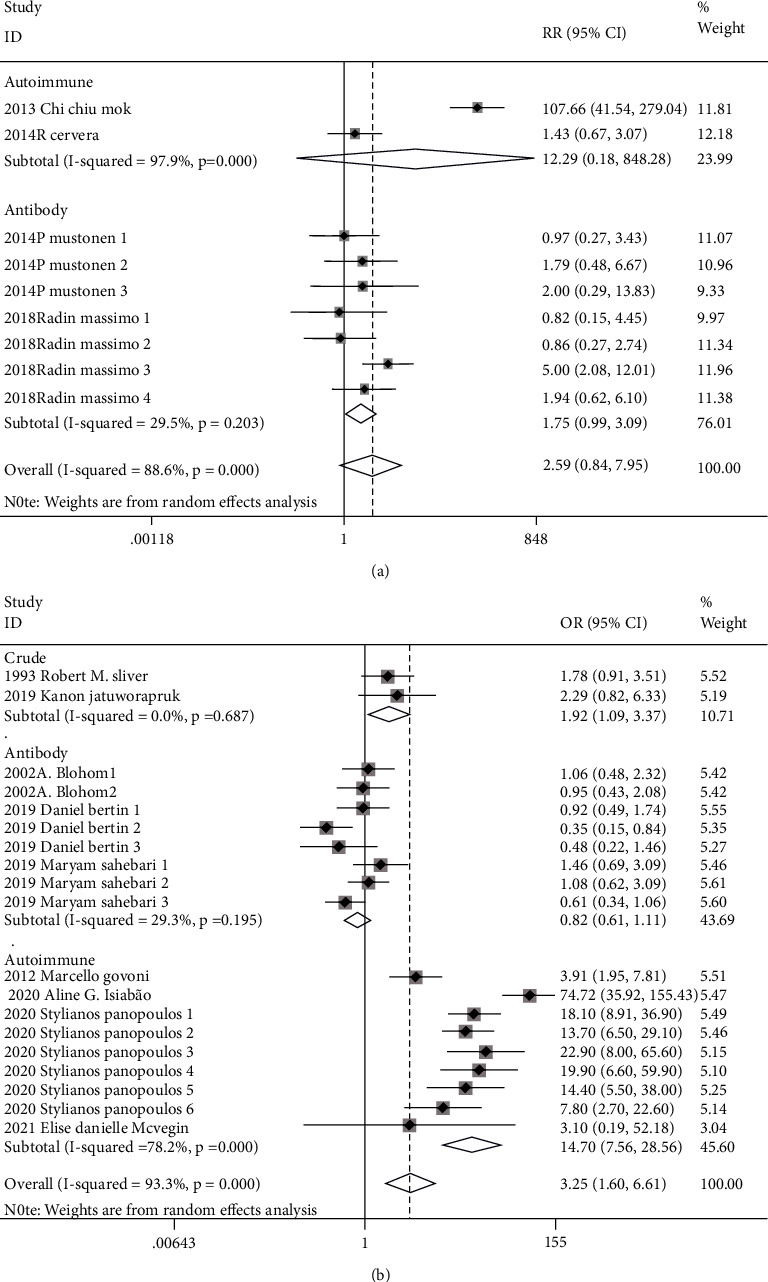
Subgroup analysis of RR/OR.

Totally pooled HR, RR, and OR were 1.76 (1.39-2.21), 2.29 (0.81-6.44), and 3.25 (1.60-6.61), respectively. Heterogenicities of totally pooled HR, RR, and OR were 0.0% (*p* = 0.547), 88.3% (*p* < 0.001), and 93.3% (*p* < 0.001), respectively (Figure 2). In sensibility analysis, after omitting the highest-quality study, the pooled HR (2018Serena Fasano), RR (2013Chi Chiu Mok), and OR (2020Aline G. Islabão) were 1.63 (1.27-2.10), 1.52 (0.97-2.38), and 2.69 (1.44-5.03), respectively. Heterogenicities of pooled HR, RR, and OR in the sensibility analysis were 0.0%, 27.8%, and 90.7%, respectively (Table 3). There were symmetrical distributions in the funnel plots of HR, RR, and OR (Figure 3). The high heterogenicities of totally pooled RR and OR might be a reason for the study's lowest quality. In the meta-analysis, after omitting those studies of HR (2016Jean-Christophe Gris), RR (2018Radin Massimo), and OR (2019Kanon Jatuworapruk), the pooled effects were 1.73 (1.13-2.63), 2.78 (0.52-14.86), and 3.32 (1.58-6.96), respectively, with still high heterogenicities, i.e., 32.5%, 92.7%, and 93.7% (Table 3), respectively. Referring to other concomitant characters of participation before studies, the subgroup analysis was performed for crude, antibody, and autoimmune in totally pooled RR and OR (Figure 4). In the subgroup analysis, the totally pooled RRs of antibody and autoimmune were 1.75 (0.99, 3.09) and 12.29 (0.18~848.28), with the heterogenicities of 29.5% (*p* = 0.203) and 97.9% (*p* < 0.001), respectively. Moreover, the totally pooled ORs of crude, antibody, and Autoimmune were 1.92 (1.09, 3.37), 0.82 (0.61-1.11), and 14.70 (7.56-28.56), with the heterogenicities of 0.0% (*p* = 0.687), 29.3% (*p* = 0.195), and 78.2% (*p* < 0.001), respectively.

## 4. Discussion

Cohort studies published in the 21^st^ century were selected herein to perform a systemic review and meta-analysis, crossing continents and involving all age and gender groups. According to definition in clinical epidemiology, HR and RR have higher qualities of explaining causal relationship, compared with OR. Moreover, time factor is involved in HR, which is an advantage of HR compared with RR [24, 25]. In our meta-analysis, the totally pooled HR supported that APS was a risk factor of stroke with low heterogenicity and a stable result of sensibility analysis. Yet the totally pooled RR showed that APS could not increase occurrence of stroke with high heterogenicity and a stable result of sensibility analysis. Totally pooled OR transmitted the same conclusion of HR with high heterogenicity and a stable result of sensibility analysis. In the subgroup analysis of totally pooled RR and OR, they both showed that the APS diagnosis absolutely based on positive antiphospholipid antibodies could not increase stroke occurrence with low heterogenicity. We considered that in patients with a purely positive antibody, who might have no characteristics in the clinic, the immune tolerance and immune response were under a balanced condition of a relatively lower sensitized immune system [26, 27]. Subgroup analysis of OR transmitted that APS patients with other concomitant autoimmune diseases or related organ injuries would have risk of stroke. Although RR had the advantage of explaining the causal relationship compared with OR, the results from the autoimmune subgroup analysis of RR with more significant heterogenicity (only enrolling two studies) might have the lower quality of evidence. In addition, the subgroup analysis of OR also showed that patients without confounding factors had risk of stroke. We considered that previous organ injuries could not only expose the location of immunologic injury via PAPS but also enlarge the injury effect of primary diseases in secondary APS via other autoimmune diseases [28, 29].

High heterogenicities represent the major limits of this study, especially for totally pooled RR and OR. However, publication bias and the lowest-quality study might not be the reason. On the contrary, omitting the study with the highest quality could reduce the heterogenicity of pooled RR. Therefore, we supposed that in the 2006 classification criteria [30], an amendment was made to the levels of antibodies for the diagnosis of APS, which could make the phenomenon that APS patients included in studies before 2006 were different from those after 2006. In addition, some unknown confounders might potentially exist in this study, such as that the participants in the nonexposed groups might receive medical prevention of stroke. In the pooled OR, we supposed that the source of heterogenicity might be from the autoimmune subgroup, which included studies with different characters of patients before the study. In particular, one study included no adults, while another study included patients suffering from RF. In addition, stroke as the definition of the endpoint might cover a wide range. We could not be sure whether ICH appears in the endpoint. In the future, studies with larger numbers of participants, longer period of observation, and more detailed grouping or definition of the endpoint in the cohort study are still needed to reduce the unknown confounders.

## 5. Conclusion

APS might be a risk factor of stroke, especially in patients with other autoimmune diseases. However, purely positive antiphospholipid antibodies might not increase the occurrence of stroke.

## Figures and Tables

**Table 1:** Characteristics of included studies.

Publication year	The first author	Countries or regions	Mean ages (SD)	Male/total participation	Other concomitant characteristics of participation before study	Period of observation (years)	Endpoint of observation
1993	Robert M. Silver	United States of America	31 (2)	19/130	N/A	10	Stroke
2002	A. Blohorn	France	37.2 (7)	69/139	2 subgroups of positive antibody types^a^	5	Stroke
2009	Kumar Rajamani	United States of America	59.2 (12.1)	292/545	Heart disease related to autoimmunity^b^	2	Stroke
2012	Marcello Govoni	Italy	46.2 (14.8)	51/469	SLE	10	Stroke
2013	Chi Chiu Mok	Hong Kong (China)	32.5 (14)	51/679	SLE	7	Stroke
2014	P. Mustonen	Finland	44.7 (14.3)	13/119	3 subgroups of positive antibody types	38	Stroke
2014	R. Cervera	Spain	42 (14)	180/552	SLE	10	Stroke
2016	Jean-Christophe Gris	France	30 (5)	0/1313	Gestation	10	TIA, ISS^c^
2018	Radin Massimo	Italy	43.4 (10.4)	5/36	4 subgroups of positive antibody types	2	Stroke
2018	Serena Fasano	Italy	37 (12.2)	17/297	2 subgroups of positive antibody types	7	Stroke
2019	Daniel Bertin	France	45 (18)	162/442	3 subgroups of positive antibody types	6	Stroke
2019	Kanon Jatuworapruk	Thailand	41.6 (17.3)	24/74	N/A	5	Stroke
2019	Maryam Sahebari	Iran	19.6 (12.46)	25/205	3 subgroups of positive antibody types	3	Stroke
2020	Aline G. Islabão	Brazil	12 (6)	231/1519	No adults	15	Stroke
2020	Stylianos Panopoulos	Greece	48.7 (13.4)	136/495	RA, SLE	14	Stroke
2021	Elise Danielle McVeigh	United States of America	38 (11.97)	9/80	SLE	17	Stroke
2021	Safifia Bano	Pakistan	28.39 (7.19)	5/50	N/A	3	Stroke

SD: standard deviation; SLE: systemic lupus erythematosus; RA: rheumatoid arthritis; TIA: transient ischemic attack; ISS: ischemic stroke. ^a^APS diagnosis was absolutely dependent on different types of positive antibodies, which meant APS patients were separated to single positive antibody group such as laC(+), aCA(+), and a*β*A(+); double positive antibody group such as laC/aCA(+), aCA/a*β*A(+), and laC/a*β*A(+); and triple positive antibody group. ^b^Apart from APS, other autoimmune diseases appeared on participation. ^c^Stroke was separated to different types: TIA, ISS, and others.

**Table 2:** Quality assessment of included studies via Newcastle-Ottawa Quality Assessment Scale Cohort Studies.

Year	Authors	Selection				Comparability	Outcome			Total
		Representative	Nonexposed	Ascertainment	Not present		Assessment	Long enough	Adequacy	
1993	Robert M. Silver	∗	∗	∗	∗	∗	∗	∗		7
2002	A. Blohorn	∗	∗	∗	∗	∗	∗	∗		7
2009	Kumar Rajamani	∗	∗	∗	∗	∗∗	∗		∗	8
2012	Marcello Govoni	∗	∗	∗	∗	∗	∗	∗		7
2013	Chi Chiu Mok	∗	∗	∗	∗	∗∗	∗	∗	∗	9
2014	P. Mustonen	∗	∗	∗	∗	∗∗	∗	∗		8
2014	R. Cervera	∗	∗	∗		∗∗	∗	∗	∗	8
2016	Jean-Christophe Gris	∗		∗		∗∗	∗	∗		6
2018	Radin Massimo	∗		∗		∗∗	∗		∗	6
2018	Serena Fasano	∗	∗	∗	∗	∗∗	∗	∗	∗	9
2019	Daniel Bertin	∗		∗	∗	∗	∗	∗		6
2019	Kanon Jatuworapruk	∗	∗	∗		∗∗	∗	∗		7
2019	Maryam Sahebari	∗	∗	∗	∗	∗	∗		∗	7
2020	Aline G. Islabão	∗	∗	∗	∗	∗∗	∗	∗	∗	9
2020	Stylianos Panopoulos	∗	∗	∗		∗∗	∗	∗		7
2021	Elise Danielle McVeigh	∗	∗	∗		∗∗	∗	∗		7
2021	Safifia Bano	∗	∗	∗	∗	∗∗	∗		∗	8

**Table 3:** Sensibility analysis of totally pooled HR/RR/OR.

Modification	*I* ^2^, HR (95% CI) (study ID)	*I* ^2^, RR (95% CI) (study ID)	*I* ^2^, OR (95% CI) (study ID)
The study with the highest quality omitted	*I* ^2^ = 0.0% 1.63 (1.27~2.10) (2018Serena Fasano)	*I* ^2^ = 27.8% 1.52 (0.97~2.38) (2013Chi Chiu Mok)	*I* ^2^ = 90.7% 2.69 (1.44~5.03) (2020Aline G. Islabão)
The study with the lowest quality omitted	*I* ^2^ = 32.5% 1.73 (1.13~2.63) (2016Jean-Christophe Gris)	*I* ^2^ = 92.7% 2.78 (0.52~14.86) (2018Radin Massimo)	*I* ^2^ = 93.7% 3.32 (1.58~6.96) (2019Kanon Jatuworapruk)
